# Supplementary material for: Unguided web-based brief intervention with genetic risk education to reduce unhealthy alcohol consumption in Japan: Protocol for a randomized controlled trial
Source: PLoS One. 2026 Apr 17;21(4):e0347064. doi: 10.1371/journal.pone.0347064 (PMC13089686; doi:10.1371/journal.pone.0347064)
Supplement: S2 Table — (DOCX) [file pone.0347064.s002.docx]

**Table S2.** BIGRE treatment video intervention script (translated from Japanese)

| **ENGLISH TRANSLATION** | **JAPANESE** |
| --- | --- |
| **NOTIFICATION THAT PARTICIPANT IS LIKELY AN *ALDH2*2* CARRIER**  Your answers to the questionnaire have identified you as being at risk for health problems if you drink alcohol. | アンケートの回答から、あなたはお酒を飲むとで健康を害しやすいことがわかりました。 |
| **BASIC INFORMATION ABOUT THE *ALDH2* ENZYME**  Do you ever turn red, feel nauseous, or feel your heart pound when you drink alcohol?  This reaction is because your body does not produce an enzyme that efficiently breaks down alcohol.  This enzyme is called ALDH2.  Alcohol enters the body and is broken down into Acetaldehyde. Acetaldehyde is like a poison and can cause cancer. ALDH2 breaks down Acetaldehyde into a non-toxic substance called Acetate.  If your body doesn’t produce enough ALDH2, the Acetaldehyde stays in your body, increasing your chances of getting cancer. | お酒を飲むと、顔が赤くなったり、吐き気をもよおしたり、心臓がドキドキしたりすることはありませんか？  この反応は、アルコールを分解する酵素が体内で十分に作られないために起きます。  この酵素は2型アルデヒド脱水素酵素（ALDH2）と呼ばれています。  アルコールは体内に入って、アセトアルデヒドに分解されます。 アセトアルデヒドは毒のようなもので、二日酔いの原因になるのですが、なんと、これが癌の原因になることもあるのです。 ALDH2はアセトアルデヒドを酢酸という安全な物質に分解します。  ALDH2が体内で十分に作られないと、アセトアルデヒドが体内に留まり、がんになる可能性が高くなるのです。 |
| **INFLUENCE OF *ALDH2* GENETIC VARIATIONS ON ALCOHOL METABOLISM**  There are 3 potential genetic variations of the ALDH2 gene that you inherit from your parents.  Type 1 is the normal type, produces an efficient ALDH2 enzyme, and allows the body to rapidly break down alcohol.  In Type 2, ALDH2 is less efficient, so you can drink alcohol, but the enzyme works more slowly and causes Acetaldehyde to accumulate after drinking.  Type 3 produces an enzyme that is completely inactive. People with this type usually get very sick after drinking small amounts and typically they do not drink alcohol at all because a high amount of Acetaldehyde accumulates.  You are probably Type 2; a type with one *ALDH2*2* allele Type 2 is more common in Japanese people than in Western countries, and about half of Japanese people fall into this type, just like you. | ALDH2には3つの遺伝子型があり、これは両親から受け継がれます。  １つ目は1型。このタイプでは、適切な量のALDH2を生成し、体内でアルコールを適切に分解することができます。  2つ目は2型。ALDH2の産生はされるので、お酒は飲めますが、それが少ないのできちんと分解されません。  3つ目は3型。ALDH2の産生がほとんどないタイプです。 このタイプの人は、一滴でもアルコールを摂取すると気分が悪くなります。つまり、アルコールが全く飲めないのです。  あなたは２型で、ALDH2*2と呼ばれるタイプです。2型は欧米に比べて日本人に多く、半分くらいはこのあなたと同じ2型に当てはまります。 |
| ***ALDH2*2* DEFICIENCY AND ESOPHAGEAL CANCER RISK ASSOCIATION INFO**  Your probable *ALDH2*2* type causes the enzyme to work less efficiently in breaking down alcohol and leaves toxic levels of Acetaldehyde in your body that increases your risk of getting cancer, especially mouth and throat cancers.  The more you drink, the greater your risk of developing cancer. | あなたはALDH2*2の遺伝子型のため、体内に危険なアセトアルデヒドが過剰に残り、がん、特に口腔がんや喉頭がんになるリスクが増加します。  お酒を飲めば飲むほど、がん発症のリスクは高まりるのです。 |
| **RISKS OF CONTINUED USE**  Doctors and researchers have found that people with the ALDH2*2 gene type (like you) who are light drinkers are nearly 2 times more likely to get mouth and throat cancer than non-drinkers. moderate drinkers are 2.4 times more likely. And heavy drinkers are 4.4 times more likely to get cancer. Some research suggests the risks are even higher than that! | 医師や研究者によれば、あなたのようにALDH2*2遺伝子を持つ人のうち、軽い飲酒をする人は飲まない人に比べてがんになる確率が約2倍、中程度の飲酒をする人は2.4倍、大酒飲みの人は4.4倍高くなります。 実際にはもっと高いリスクがあると指摘する研究者もいます。 |
| **BENEFITS OF REDUCING USE**  The easiest way to reduce your risk of getting mouth and throat cancers associated with alcohol is to reduce the amount of alcohol you drink and your exposure to Acetaldehyde. Obviously, less alcohol is better for your health. Drinking less alcohol can have other added health benefits. | アルコールに関連する口や喉のがんになるリスクを減らす最も簡単な方法は、お酒の量を減らすことです。もちろん、アルコールの量が少ない方が健康には良いのです。お酒の量を減らすことは、他の健康上の利点もあります。 |
| **ADVICE ABOUT HOW TO REDUCE USE**  The best way to reduce your alcohol use is to think about what is important to you. Is it your spouse, children, or grandchildren? Is it your career? Maybe it’s your spiritual beliefs?  Think about what is important to you and about how health problems like cancer would stop you from living the life you want to live.  You don’t need to cut out all alcohol. But remember, the more you drink, the greater your risk for cancers and other alcohol-related health problems. | アルコールの使用を減らす最善の方法は、自分にとって何が大切かを考えることです。それは配偶者、子供、孫と過ごす時間でしょうか？充実した仕事でしょうか？気持ちのゆとりでしょうか？  自分にとって何が大切なのか、そして、癌のような健康上の問題によって、自分が生きたい人生を送ることがいかにできなくなるか、を考えてみてください。  アルコールをすべて断つ必要はありません。 しかし、お酒を飲めば飲むほど、リスクが高まる、そのことを忘れないでください。 |
| **DEVELOPING A PLAN TO REDUCE USE**  If you think reducing your alcohol consumption might be a good idea, let’s create a plan.  First, decide how ready and committed to changing you are. Even if it’s just a little, that’s pretty great.  Next, try to change the way you think and talk. Instead of saying things like, “I might want to reduce alcohol,” try using language that is stronger. Something like, “I will cut down on drinking,” which is a better way to plan.  Next, let’s set some goals. You should decide how much you want to cut down. Be specific: “I will drink 3 fewer beers per day or 10 fewer go of sake per week.”  Then, identify any barriers to your goal that will hurt your success. Do you have a friend who makes it easy to drink? Are there izakaya where you tend to drink more? Would buying less otsumami help? Hey, reducing salt intake, which might make you want to drink more, might not be bad either. | 飲酒量を減らした方がいい、そう思い立ったら、計画を立ててみましょう。  まず、自分がどの程度の覚悟と決意を持っているのかをはっきりさせましょう。ほんの少しであっても、それはとても素晴らしいことです。  次に、考え方や話し方を変えてみましょう。「お酒を控えようかな」ではなく「お酒を減らす」などと、より強い言葉で表現してみましょう。  次に、目標を設定しましょう。 どのくらい減らしたいかを決めましょう。 具体的には、「1日に飲むビールを3本減らす」「1週間に飲む日本酒を10合減らす」などです。  そして、目標にに立ちはだかる壁を明らかにすることが、成功への近道となります。 友人でお酒を飲みやすい人はいますか？ ついつい飲んでしまう居酒屋はありますか？ おつまみを買う回数を減らせばいいのかも？ 塩分を減らすのも悪くないかもしれませんね。 |
| **Close**  If you are concerned about your health, sometimes a little motivation can help a lot.  Remember, your ALDH2*2 genetics put you at a greater risk for some cancers, depending on how much you drink. Reducing your alcohol just a little can help, and the more you reduce your alcohol consumption, the lower your risk.  Good luck. | もしあなたが健康を気にかけているなら、ちょっとした心がけで全てが変わることもあります。  ALDH2が2型のあなたは、飲酒量に応じて、がんのリスクが高くなることを覚えておいてください。アルコールを少し減らすだけで、そのリスクを減らすことができるのです。  一緒にがんばりましょう。 |
