# Supplementary material for: Unguided web-based brief intervention with genetic risk education to reduce unhealthy alcohol consumption in Japan: Protocol for a randomized controlled trial
Source: PLoS One. 2026 Apr 17;21(4):e0347064. doi: 10.1371/journal.pone.0347064 (PMC13089686; doi:10.1371/journal.pone.0347064)
Supplement: S3 Table — (DOCX) [file pone.0347064.s003.docx]

**Table S3.** QoL Sham control video script (translated from Japanese)

| **ENGLISH TRANSLATION** | **JAPANESE** |
| --- | --- |
| Have you ever heard the term Quality of Life (QOL)?  It's not simply about being free from illness, but about being physically, psychologically, and socially healthy.  It has been found that improving this QOL has many benefits. | 生活の質（QOL）って言葉を聞いたことはありますか？  単に病気がないということではなく、身体的にも、心理的にも、社会的にも健康でいることです  このQOLを向上させることには多くの利点があることが分かりました。 |
| Have you been having trouble sleeping at night or feeling depressed or anxious lately?  This can be associated with a decreased quality of life.  Furthermore, it has been shown that a lower quality of life can lead to lower efficiency in learning and working. | 最近、夜寝付けなかったり、抑うつ気分や不安を感じたりすることはありませんか？  これは、生活の質(QOL)の低下につながります。  更に、QOLが低下すると、学習や労働の効率が低くなることもわかっています。 |
| Quality of life is assessed by the WHOQOL-BREF, which measures four domains with a 26-item scale.  The first domain is physical health. It is related to the presence or absence of illness, physical pain, or discomfort.  The second domain is mental state. It involves whether you enjoy life, accept your appearance, and are satisfied with your ability to do your job.  The third domain is social. It concerns whether you are satisfied with your relationships, your sex life, and the support of your friends.  The fourth domain is environmental. It is related to your accessibility to medical facilities, social services, and whether you are satisfied with your surrounding transportation system. | QOLは、４つの領域を26項目のアンケートで「WHOQOL-BREF」によって評価します。  １つ目は身体的健康。病気や体の痛み、不快感の有無に関するものです。  ２つ目は心の状態。生活を楽しんでいるか、自分の容姿を受け入れることができるか、自分の仕事をする能力に満足しているかなどといったことが関係します。  ３つ目は人とのつながり。人間関係、性生活、友人たちの支えに満足しているかが関係します。  ４つ目は環境。医療施設や福祉サービスの利用しやすさ、周囲の交通の便に満足しているかが関係します。 |
| A better quality of life means a healthier and more satisfied body and mind. Plus, quality of life gives you a greater ability to enjoy life. | QOLを高めると、心身ともに健康で満足した状態になり、人生を楽しむことができるようになります。 |
| Lower quality of life can lead to sleep disturbances, depressed mood, anxiety, and decreased work productivity. | QOLが低いと、睡眠障害が起きたり、抑うつ気分や不安に陥ったり、仕事の生産性が低下したりします。 |
| There are countless ways to improve quality of life.  For example, you can take a long bath, take deep breaths, go out, or meet with other people.  For example, you can reflect on what you accomplished today, plan your life, or watch people.  For example, you can take an active role in community activities, take up a new sport, or help others.  You may also want to just sit and think about things or practice mindful contemplation. | QOLを高める方法は無数にあります。  例えば、お風呂にゆっくりつかったり、深呼吸をしたり、出かけてみたり、人と会ったり。  例えば、今日１日自分がやり遂げたことを思い返してみたり、人生設計を立てたり、人間観察をしたり。  例えば、地域の活動に積極的に参加したり、新しいスポーツを始めたり、人助けをしてみたり。  ただ座って考えごとをするのもいいかもしれません。 |
| The best way to change something to improve the quality of life is to think about what is important to you. Is it your spouse, children, or grandchildren? Is it your friends? Is it your career? Maybe it’s your spiritual beliefs?  Think about what is important to you and about how health and mental problems would stop you from living the life you want to live.  You don’t need to make a whole change. But remember, if you do not make changes at all, you yourself will not change at all. | 生活の質を高めるために何かを変えるには、自分にとって何が大切かを考えるのが一番です。それは配偶者でしょうか、子供でしょうか、孫でしょうか？友人でしょうか？キャリアでしょうか？ もしかしたら、自分の心の奥にある信念かもしれません。  自分にとって何が大切なのか、そして健康や心の問題によって、自分が生きたい人生を送ることがいかにできなくなるか、を考えてみてください。  全部を変える必要はありません。 しかし、何も変えなければ、あなた自身も何も変わらないことを忘れないでください。 |
| If you think doing something to improve the quality of life might be a good idea, let’s create a plan.  First, decide how ready and committed to changing you are. Even if it’s just a little, that’s pretty great.  Next, try to change the way you think and talk. Instead of saying things like, “I might do ***,” try using language that is stronger. Something like, “I will ***,” is a better way to plan.  Next, let’s set some goals. You should decide how much you want to make changes. Be specific: “I will take a bath for 30 minutes, take a deep breath once a day, or go on a gym twice a week.”  Then, identifying barriers to your goal will help you be successful. Does skipping breakfast prevent you from being active? Is the cost of joining a sport club an obstacle? Would searching sports you can do near your house help? Hey, thinking of doing something at home might not be bad either. | 生活の質を高めるために何かした方がいい、そう思ったら、計画を立ててみましょう。  まず、自分がどの程度の覚悟と決意を持っているのかをはっきりさせましょう。ほんの少しであっても、それはとても素晴らしいことです。  次に、考え方や話し方を変えてみましょう。「〇〇しようかな」ではなく、「私は○○する」というような、より強い言葉で計画を立ててみましょう。  次に、目標を設定しましょう。 どの程度変化させたいかを決めるとよいでしょう。 具体的には、「30分お風呂に入る」、「1日1回深呼吸をする」、「週に2回ジムに通う 」などです。  そして、目標に立ちはだかる壁がわかっていると、うまくいく助けになります。朝食を抜いているために、元気になれないことなないでしょうか？ スポーツクラブに入会するための費用が障害になっていますか？ 家の近くでできるスポーツを探す方がいいのかも？ 家でできることを考えるのも悪くないかもしれませんね。 |
| You don't have to do it for others. You can do what you enjoy doing. | 人のためにする必要はありません。自分が楽しいと思うことをしましょう。 |
| Just try a few new things, it can really help improve your quality of life. | ちょっとした新しいことを試してみて、すると生活の質はよくなるはずです。 |
