# Supplementary material for: Unguided web-based brief intervention with genetic risk education to reduce unhealthy alcohol consumption in Japan: Protocol for a randomized controlled trial
Source: PLoS One. 2026 Apr 17;21(4):e0347064. doi: 10.1371/journal.pone.0347064 (PMC13089686; doi:10.1371/journal.pone.0347064)
Supplement: S4 Table — (DOCX) [file pone.0347064.s004.docx]

**Table S4.** Ethics Review Submission **(**translated from Japanese)

**Intervention Study Protocol**

**Brief Intervention to Promote Health Behaviors: A Randomized Controlled Trial**

**Principal Investigator:** Ethan Sahker

**Affiliation:**
Graduate School of Medicine, Kyoto University
Department of Health Promotion and Behavioral Science
Center for Medical Education and Internationalization
Population Health Policy Research Unit

This study protocol contains confidential information and is provided only to individuals and organizations involved in the conduct of this study, including implementing medical institutions (principal investigator, co-investigators, research participants), the ethics review committee, the efficacy and safety evaluation committee, and other relevant personnel.
Except when explaining the study to research participants, this study protocol must not be disclosed to any third party or used for any purpose other than conducting this study without written consent from the principal investigator.

**Protocol Number:** Kyoto University Graduate School of Medicine Ethics Committee (C1711-2)

**Trial Registration Number:** UMIN000058012
**Version 0.1.2025.1.29**

**1. Title of the Study**

Brief Intervention to Promote Health Behaviors: A Randomized Controlled Trial

**2. Background of the Study**

Alcohol consumption is the third leading cause of global disease burden [1] and a major risk factor for cancer [2]. Among all alcohol-related cancers, esophageal cancer has the highest proportion attributable to alcohol use [3]. In other words, esophageal cancer risk is reduced more than any other alcohol-related cancer when alcohol consumption stops. Importantly, reducing or quitting alcohol consumption significantly decreases esophageal cancer risk [4]. In Japan, the burden of esophageal cancer attributable to alcohol is substantial, with an estimated 5,279 deaths and 102,988 disability-adjusted life years annually [5].

Acetaldehyde—an established carcinogenic metabolite of alcohol—is the causal factor in alcohol-related esophageal cancer [5]. Genetic variations in alcohol metabolism are known to influence esophageal cancer risk [6]. The *ALDH2* gene encodes the primary enzyme responsible for acetaldehyde metabolism. *ALDH2* shows the strongest association with esophageal cancer risk. The *ALDH2*2* allele leads to reduced enzyme activity, impairing acetaldehyde metabolism [7].

Thus, individuals with *ALDH2* deficiency (carriers of the *ALDH2*2* allele*)* experience elevated acetaldehyde exposure during alcohol consumption, resulting in “Asian flush,” a common reaction among Japanese people [6,8]. Although *ALDH2*2* is generally negatively correlated with alcohol use disorder [9], many carriers still engage in moderate to heavy drinking [6].

The prevalence of *ALDH2*2* is high among Japanese individuals, estimated at 41–52% [10]. As alcohol consumption increases, those with *ALDH2*2* show a clear dose–response increase in esophageal cancer risk [6], and this increase is substantially larger than in non-carriers.

A meta-analysis showed that compared with other genotypes, *ALDH2**2 carriers have:

- 2.49× higher risk at moderate drinking (CI: 1.29–4.79)
- 7.07× higher risk at heavy drinking [11]

Additional studies not included in the meta-analysis showed even higher odds ratios when comparing *ALDH2**2 non-drinkers with moderate drinkers (OR = 9.64–52.86) and heavy drinkers (OR = 77.1–95.4) [12,13]. Because Japan has a particularly high prevalence of *ALDH2**2, the alcohol-attributable burden of esophageal cancer is estimated to be twice the global estimate, suggesting that reducing alcohol consumption among *ALDH2**2 carriers could substantially decrease disease incidence [14]. Nevertheless, these associations remain poorly recognized [6].

Effective brief interventions (BIs) for alcohol use exist [15], providing an opportunity for primary prevention among individuals with high genetic risk for alcohol-related cancers. BIs are typically delivered in single sessions lasting 5–60 minutes and are effective for low-to-moderate alcohol use [16]. BIs are based on social, cognitive, and behavioral therapeutic models [15]. Key BI components include: Feedback about alcohol use and harms, Risks of continued use, Benefits of reducing use, Advice on reduction strategies, Motivational enhancement, Planning for reducing use. These components follow the FRAMES approach (Feedback, Responsibility, Advice, Menu of options, Empathy, Self-efficacy) [15,18–20] and the stages of change model [17].

Recent evidence shows that BIs effectively reduce alcohol consumption among Asian populations. In the U.S., a pilot study adapted a web-based BI incorporating ALDH2 cancer-risk information for Asian American college students, demonstrating feasibility, acceptability, and effectiveness for *ALDH2**2 carriers [21]. A study among Japanese university students also found that a BI using *ALDH2**2 cancer-risk information significantly reduced alcohol consumption and increased motivation to change compared with controls [22]. However, that study required genetic testing using blood samples and involved guided, in-person or remote intervention delivery.

Using a simple, non-invasive ALDH2 screening method with an unguided web-based BI may be a cost-effective approach for reaching individuals reluctant to seek treatment [23,24]. Such an intervention could reduce healthcare burden, but its usefulness in Japan remains unproven.

This study investigates whether a non-invasive, unguided, web-based BI using personalized genetic risk education (BIGRE) is effective in reducing alcohol consumption among Japanese individuals. If effective, this approach could be widely disseminated in clinical and public health settings or via public service announcements. Guided by principles of personalized medicine and public health genomics [22], the primary objective is to determine whether web-based genetic risk education reduces alcohol consumption compared with a control group among Japanese adults who screen positive for heavy drinking and possible *ALDH2**2 genotype. Secondary outcomes examine subgroup differences in consumption, severity, motivation, risk knowledge retention, and QOL.

**3. Objectives and Significance of the Study**

The purpose of this study is to examine the effectiveness of a web-based brief intervention (BI) for individuals who exceed the alcohol intake levels recommended by the Ministry of Health, Labour and Welfare and who are likely carriers of the *ALDH2**2 allele. If the BI proves effective, it may not only reduce alcohol-related healthcare costs but also contribute to addressing alcohol-related family and social issues. Furthermore, a web-based BI can be incorporated into public health activities in clinics and hospitals and can support public messaging through service announcements targeted at younger populations with alcohol-related risks.

**4. Participant Selection Criteria**

**Setting**

This study will be conducted online. Recruitment will be carried out using social media, community outreach, and medical advertisements.

**Eligibility Criteria**

***Inclusion criteria***

Participants must meet all of the following criteria (1–6):

1. Age between 20 and 64 years
2. Able to read, write, and understand Japanese
3. Consumed 14 or more standard drinks in the past 4 weeks
4. Positive *ALDH2**2 screening result
5. Has access to an internet connection
6. Owns a current email account

One standard drink = 10 g of pure alcohol. Alcohol consumption frequency will be evaluated using the Japanese version of the Daily Drinking Questionnaire (DDQ). Details are provided in below. ALDH2 heterozygous or homozygous deficiency will be screened using the simplified flushing questionnaire, which has high sensitivity (90%) and specificity (88%) for alcohol-related flushing response. A “Yes” response to either question indicates likely ALDH2 deficiency.

Screening questions:

a) “Do you currently experience facial flushing after drinking a small amount of alcohol such as one beer?”

b) “During your first 1–2 years of drinking, did you experience such flushing?”

Rationale for Inclusion Criteria

1. Individuals aged 65 or older generally require reduced alcohol intake due to decreased metabolic function; therefore, they are excluded.
2. Items 2, 5, 6 ensure that participants can complete the online Japanese-language protocol.
3. According to the Ministry of Health, Labour and Welfare’s “Health Japan 21 (First Term),” appropriate drinking is defined as an average of 20 g of pure alcohol per day for Japanese adults with normal metabolic capacity. Since 1 drink = 10 g, 14 drinks/week is considered the upper limit. Consumption above this threshold is classified as harmful drinking.

***Exclusion criteria***

Participants will be excluded if any of the following apply (1–3):

1. Currently receiving treatment for alcohol dependence or alcohol-related disorders
2. Has a history of cancer diagnosis or treatment
3. Pregnant

Rationale for Exclusion Criteria

1. Individuals already in treatment for alcohol-related conditions may reduce alcohol use independently of BI due to treatment requirements.
2. Individuals with prior cancer may perceive cancer-risk information differently than those without such history, which could bias outcomes.
3. Pregnant individuals are typically advised to avoid alcohol regardless of BI.

**Planned Sample Size and Rationale**

The target sample size is 100 participants. Based on allele frequency estimates for Japanese populations [10], 41–52% are expected to carry the ALDH2*2 allele. Prior studies indicate that individuals consuming ≥14 standard drinks over the past 30 days may exhibit a 50% reduction following the intervention [21]. Sample size calculations indicate that 64 participants (32 per group) provide 80% power to detect a 7-drink difference (SD=10) at follow-up (α=0.05, β=0.20).

To account for attrition, the target was increased to 100 participants.

**5. Study Methods and Scientific Rationale**

**1) Design**

- Study phase: **Confirmatory study**
- Comparison: **Parallel-group comparison**
- Control type: **Placebo control**
- Allocation: **Randomized**
- Blinding: **Triple-blinded** (including evaluator blinding)

**2) Methods**

**Participant Registration**

Recruitment will be conducted through online advertisements, community outreach, and medical advertisements. Neo Marketing Co., Ltd. will support recruitment using a research panel representative of the Japanese population and will manage screening and intervention delivery via a web-based portal. Participants will be screened through the research panel, with communications conducted via email and the LINE app. Those who meet all eligibility criteria will be asked to provide informed consent. After consent, participants will be randomly assigned to one of two groups:

1. Intervention group: Unguided BI with genetic risk education (BIGRE)
2. Control group: Placebo quality-of-life (QoL) health information (QoL Control)

Through the web portal:

- Intervention group participants will watch a 6-minute genetic risk education video explaining *ALDH2**2-related cancer risks.
- Control group participants will watch a 5-minute placebo video on QoL and general health.

Time required for screening, evaluation, and intervention is 30–45 minutes.

Follow-up contacts will occur 1, 2, and 3 months after randomization via email, telephone, or LINE.
Follow-up assessments include alcohol intake questions and recall of ALDH2 genotype information.

Figure 1 illustrates the recruitment, consent, registration, and allocation flow.


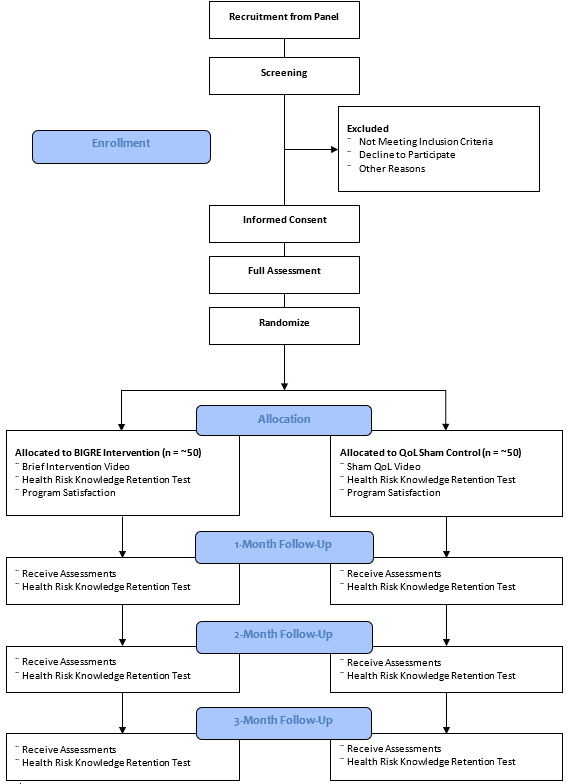


**Figure 1.** CONSORT diagram for the web-based screening and brief intervention with genetic risk education to reduce alcohol consumption.

**Allocation**

Participants will be randomized 1:1 immediately after screening, consent, and baseline assessment.
Randomization will be conducted by Neo Marketing Co., Ltd. using a centralized computer program.

Assignments will occur automatically through the survey system, ensuring allocation concealment.

Interventions (actual or placebo) begin immediately after randomization.

**Blinding Procedures**

The principal investigator will be blinded to allocation until all interventions are completed. Participants will be told the study examines “health behaviors,” without specifying alcohol BI, ensuring participant blinding. Automated educational videos provide the intervention; thus, the intervention provider is technically blinded. Outcomes are based on self-report questionnaires, preserving evaluator blinding. Data analysts will be blinded until all analyses and interpretations are completed.

**Intervention Group**

The BI will include:

1. Basic information about the ALDH2 enzyme
2. Effects of ALDH2 genetic variation on alcohol metabolism
3. Feedback indicating participant’s likely ALDH2*2 carrier status
4. Information linking *ALDH2**2 deficiency with esophageal cancer risk
5. Risks of continuing alcohol use
6. Benefits of reducing use
7. Motivational enhancement strategies
8. Planning for reducing use
9. Empathic delivery supporting self-efficacy through genetic education

Key messages emphasized:

1. *ALDH2*2* carriers experience increased cancer risk, which rises with alcohol intake.
2. *ALDH2*2* is not determinative—this is not a diagnostic test.
3. ALDH2*2 carriers who abstain or drink infrequently do not show elevated cancer risk.
4. Video length: 5–7 minutes, using graphical content.

**Control Group**

Control group participants will receive a sham educational video explaining:

1. Importance of quality of life (QoL)
2. Definitions of QoL domains
3. Health behavior changes that support QoL

This sham video features a physician avatar with professional narration and 5–7 minutes of audio and animation.

**3) Observations, Tests, Assessments, and Schedule**

**Measures, Methods, Assessors, and Timing**

| **Measure** | **Baseline** | **Follow-Up** |
| --- | --- | --- |
| Participant background | ✔ |  |
| **Primary Outcome** |  |  |
| DDQ | ✔ | ✔ |
| **Secondary Outcomes** |  |  |
| AUDIT | ✔ | ✔ |
| WHOQOL-BREF | ✔ | ✔ |
| Health-risk knowledge retention | ✔ | ✔ |
| Satisfaction with intervention | ✔ | ✔ |
| Qualitative comments | ✔ | ✔ |

**Participant Assessment Schedule**

Assessments will be conducted at:

1. Baseline
2. 1 month after intervention
3. 2 months after intervention
4. 3 months after intervention

These assessments will evaluate the intervention’s effects and changes over time.Neo Marketing Co., Ltd. will guide participants through the LINE app to the portal site, where they will complete a 47-item online questionnaire.

Estimated time required:

1. Baseline assessment + intervention: 30–40 minutes
2. Each follow-up assessment: ~15 minutes

**4) Overview of Analysis**

**Primary Outcome: *Alcohol consumption frequency***
Measured as the number of standard drinks consumed in the past 30 days at 1-, 2-, and 3-month follow-ups. Definition of one standard drink (approximate):

1. Beer: 340 ml (~5% alcohol)
2. Wine / Sake: 100 ml (~13% alcohol)
3. Highball/Collins: 250 ml (45 ml whisky)

Alcohol use is measured using the Japanese version of the Daily Drinking Questionnaire (DDQ) [23]. The DDQ asks: “Thinking about a typical week in the past 3 months, how many alcoholic drinks do you consume on each day of a typical week?” Participants report consumption for each day, then totals are used to calculate a 30-day average. The DDQ has been used among Asian populations with the ALDH2*2 allele [21], showing: good internal validity (Cronbach’s α = 0.83) [24], adequate test–retest reliability [25], criterion validity [26]

**Secondary Outcomes**

***Severity of Alcohol Misuse***

Assessed using the Japanese version of the Alcohol Use Disorders Identification Test (AUDIT).

AUDIT: [30]

1. 10 items
2. Score range: 0–40
3. Screens from no/low risk to severe misuse [27]

Psychometric evidence:

1. High sensitivity (0.90s) and specificity (0.80s) at cutoff = 7 [27]
2. High test–retest reliability (K-S = 0.80s) [28]
3. Moderate stability for severity screening (ICC = 0.56, 95% CI = 0.52–0.60) [29]
4. Japanese version: acceptable internal consistency (α = .67)

***Retention of Health-Risk Knowledge***

Assessed using three items:

1. “Do you remember the genotype associated with your alcohol risk?”
2. “Does this genotype increase or decrease esophageal cancer risk when you drink alcohol?”
3. “How does alcohol consumption influence your risk of being diagnosed with cancer?”

***Quality of Life (QoL)***

Measured using the Japanese version of the WHOQOL-BREF, which evaluates QoL in four domains:

1. Physical health
2. Psychological health
3. Social relationships
4. Environment

Psychometric evidence for Japanese samples:

1. Cronbach’s α = 0.66–0.75
2. Good discriminant validity (p < .01)
3. International α range = 0.68–0.82

***Participant Satisfaction with Program***

Rated with five Likert-type items (strongly disagree → strongly agree):

1. “Program information was important to me.”
2. “This program made me reflect on my lifestyle.”
3. “I found the program interesting.”
4. “I understood the program content.”
5. “I would recommend this program to others.”

***Qualitative Comments***

Collected using an open-ended prompt: “Please share any opinions or suggestions you have about the video.”

**Other: Background Variables**

1. Sex
2. Age
3. Educational background
4. Prefecture of residence
5. Household size
6. Status of treatment for addiction

**5) Main Analysis Methods**

Based on established allele frequencies in Japanese populations [6], 41–52% are expected to carry ALDH2*2. Sample size calculations indicate that 64 participants (32 per group) provide 80% power to detect a mean group difference of 7 standard drinks (SD=10) at follow-up (p < 0.05).
Considering attrition, the target is 100 participants.

Analysis Sets:

**Full Analysis Set (FAS)**
Includes the largest feasible sample under the intention-to-treat principle; excludes participants who withdraw consent for data use or have no outcome data.

**Safety Analysis Set (SAS)**
Includes all randomized participants, including those who discontinue or drop out.

**Primary Outcome Analysis**

The FAS will be used.
A generalized linear mixed model (GLMM) assuming a negative binomial distribution will compare intervention vs. control for alcohol consumption (DDQ) at 3-month follow-up.

Model includes:

1. Fixed effects:
   1. Group (intervention vs. control)
   2. Time (1, 2, 3 months)
   3. Group × Time interaction
2. Random intercept: participant
3. Outputs include estimated marginal means (EMM) and 95% CI at 3 months.

**Secondary Outcomes (Continuous Variables)**

Analyzed using Mixed-Effects Models for Repeated Measures (MMRM):

Covariates include:

1. Group
2. Time
3. Group × Time interaction

If convergence fails due to small sample size, time will be treated as a continuous variable assuming linear change.

**Additional Secondary Analyses**

1. DDQ scores at 1 and 2 months: GLMM
2. Moderation analysis predicting endpoint DDQ: GLMM
3. Alcohol consumption (grams): MMRM
4. Motivation to change: MMRM
5. QoL: MMRM
6. Knowledge retention: logistic regression
7. Participant satisfaction: descriptive statistics

Alpha will be set to *p* < 0.05 (two-sided), and effect sizes will be reported as Standardized Mean Difference (SMD) and Number Needed to Treat (NNT). All analyses will use RStudio version 2024.12.0 Build 467.

**6) Research Period**

1. Research Participant Registration Period: From the implementation approval date by the head of the research institution to March 31, 2028
2. Research Participant Observation Period: From the registration date to the assessment approximately 3 months later
3. Research Implementation Period: From the implementation approval date by the head of the research institution to March 31, 2029

**7) Procedures for Obtaining Informed Consent (hereinafter, “IC”)**

In this study, IC will be obtained electronically when collecting information from research participants.

1. Electronic Consent
   Neo Marketing will be responsible for confirming consent to participate in the study and verifying personal identity. Identity verification will be performed by requesting participants to submit a photo ID, such as a driver’s license, when they are guided to the LINE app.
2. Re-consent Following Amendments to the Research Protocol
   If amendments to the research protocol occur, a follow-up question regarding consent will be posted on the portal website as needed, and consent will be reconfirmed using the same procedure.

**8) Handling of Personal Information**

**Types of Personal Information Used in the Study**
Information in which participant names are converted into IDs (personal information)

**Timing and Method of Creating IDs**
ID conversion will be performed immediately after enrollment and conducted by Neo Marketing.

**Items of Personal Information to Be Retained or Used, Safety Management Measures, and Precautions**

1. Organizational Safety Measures: Limit researchers handling personal information.
2. Human Safety Measures: Researchers undergo regular training based on university regulations.
3. Physical Safety Measures: Hard drive storage areas will be locked with physical keys.
4. Technical Safety Measures: Prevent unauthorized external access.

**Person Responsible for Information Management Across the Entire Research Organization**
Name: Ethan Sahker
Affiliation: Kyoto University Graduate School of Medicine, Department of Social and Behavioral Sciences, Health Promotion and Behavior
Kyoto University Graduate School of Medicine, Center for Medical Education / Population Health Policy Unit

**Handling of Data After Withdrawal of Consent**
Until the intervention is completed, data will be deleted by Neo Marketing. After analysis begins, data will be deleted by our research office.

**9) Burden, Anticipated Risks, Benefits, Overall Assessment, and Mitigation Measures**

**Burden and Risks**
Psychological and time-related burden may arise from participating in health education and completing questionnaires. However, because all intervention procedures occur online and screening through intervention require approximately 30–45 minutes, participant burden is considered minimal. Therefore, this intervention is considered a minor invasion with no expected serious health risk.

**Benefits**
Previous studies investigating alcohol use, the ALDH2*2 gene, and cancer risk have reported no adverse events and high levels of satisfaction, interest, and engagement among participants [21]. Therefore, similar benefits may occur in this study. However, specific benefits to participants in this study are currently unknown.

**Overall Evaluation of Burden/Risks and Benefits**
Based on the above two points, this study is considered likely to provide benefits to participants. All risks and benefits will be fully disclosed and explained to potential participants during the IC process.

**Measures to Minimize Burden and Risk**
Participants will receive compensation for participating. Health risks are considered negligible.

**10. Storage and Disposal of Samples and Information**

**Storage Period for Samples and Information**
Data will be stored for 10 years after publication of the primary results.

**Storage Methods (including prevention of leaks, contamination, theft, loss, etc.)**
Data will be collected and stored by Neo Marketing. Participants must agree to the company’s data storage policies before enrolling. Data will be stored under the company’s personal information protection policies. Three months after the end of the study, data will be deleted. After data collection is complete, the company will provide the principal investigator with unprocessed data with protected medical information removed. Participants will receive the intervention through the LINE app; each participant will be assigned a unique ID allowing removal from analysis upon request. These data will be stored on password-protected secure servers in locked facilities at Kyoto University. Only the principal investigator will have access.

**Disposal Method After the Storage Period**Upon completion of the study, server-stored data will be permanently deleted. Downloaded or media-stored data will be kept for 10 years after publication of primary results and then disposed of in a manner preventing personal identification.

**Records of Provision or Receipt of Samples/Information to/from Other Institutions**No information collected in this study will be provided to researchers at other institutions.

**11. Secondary Use of Samples/Information and Possibility of Provision to Other Research Institutions**

Samples/information collected in this study may be used for future research not yet specified at the time of consent. Secondary use or provision to other institutions will occur only after approval by an ethics review committee for the new research plan. The study purpose and related information will be publicly disclosed on a website, and participants will be given the opportunity to opt out.

Website URL: <https://sahkerlabhealth.org>

**12. Reports to the Ethics Committee and the Head of the Research Institution**

The Principal Investigator (PI) will report study information in writing according to regulations.
Annual reports will be submitted once every 3 years or more. Suspension/termination reports will be submitted as needed.

- 1. If revisions are made to the research protocol, the PI will report the changes to obtain approval from the head of the institution.
  2. If unexpected serious adverse events or new information affecting study continuation occur, the PI will report promptly.
  3. If information is obtained that may compromise the appropriateness of study conduct or the reliability of results, reporting will occur promptly.
  4. If information is obtained that may compromise ethical or scientific validity, reporting will occur promptly.

**13. Research Funding and Conflicts of Interest**

**Type and Provider of Research Funding**
Japan Society for the Promotion of Science, Grant-in-Aid for Scientific Research
Project title: *Randomized controlled trial using genetic risk information to promote short-term intervention for reducing alcohol consumption*
Category: Early-Career Researchers
Grant #24K20239

**Relationship Between Provider and Researchers / Involvement in Study Design, Conduct, Analysis, or Manuscript Preparation**日本学術振興会 is not involved in the study's planning, analysis, or manuscript writing.

**Conflicts of Interest**Conflicts of interest are reviewed appropriately in accordance with Kyoto University COI Policy and COI Management Regulations by the Kyoto University Clinical Research COI Committee.
Neo Marketing, a collaborating organization, will oversee participant recruitment.

**14. Method of Public Disclosure of Study Information**

The study will be preregistered on ClinicalTrials.gov and updated accordingly. This research protocol will be published in an English-language academic journal. A Japanese-language summary will be posted on the website of Kyoto University Graduate School of Medicine, Department of Health Promotion & Behavioral Sciences. Results will be disseminated through academic journals and conferences. A Japanese-language summary of findings will also be posted online to ensure accessibility for participants. Authorship for primary and secondary outcomes will include all members of the Kyoto University Center for Medical Education and Internationalization and appropriate contributors. Authorship order will be determined based on contribution.

**15. Consultation for Research Participants and Their Contacts**

Study-specific Contact Point: Yuki Kono, 5th-year medical student, Kyoto University
Email: kono.yuki.57k@st.kyoto-u.ac.jp

Kyoto University Contact for Complaints: Kyoto University Graduate School of Medicine, General Affairs & Planning, Research Promotion Section
Tel: 075-753-9301
Email: 060kensui@mail2.adm.kyoto-u.ac.jp

**16. Participant Financial Burden and Compensation**

Participants who complete the initial screening, baseline survey, and intervention will receive a gift card worth 700 yen. Participants who complete the 1-, 2-, and 3-month follow-up surveys will receive an additional 200 yen for each. Participants completing all procedures will receive an additional 1,000 yen (maximum total: 2,300 yen). The study is conducted online; participation and follow-up assessments are via survey only. No clinical testing or clinic visits are required. Participants are responsible for their own communication costs.

**17. Handling of Research Results (Including Incidental Findings)**

Genetic information obtained in this study is based on simple self-report and is publicly accessible online. Results will be fed back directly to participants; however, it is highly unlikely that findings will relate to significant hereditary traits or health risks. Genetic counseling is not considered necessary.

**18. Research Team Structure**

**Principal Investigator**
Name: Ethan Sahker
Affiliation: Kyoto University Graduate School of Medicine, Department of Social and Behavioral Sciences, Health Promotion and Behavior
Kyoto University Center for Medical Education and Internationalization, Population Health Policy Unit
Roles: Study oversight, planning, conduct, manuscript preparation

**Co-investigators**
Yan Luo: Assistant Professor, Kyoto University Center for Medical Education and Internationalization / Population Health Policy Unit (responsible for analysis)
Yuki Kono: 5th-year medical student, Kyoto University (responsible for study operations)
Toshi A. Furukawa: Professor (Special Appointment), Kyoto University Institute for Strategic Planning (responsible for study operations)

**Collaborating Organization**
Neo Marketing (Facility Director: Yoshiki Sakajiri), responsible for participant recruitment and intervention delivery.

**Person Responsible for Sample/Information Management**
Name: Ethan Sahker
Affiliation: Kyoto University Graduate School of Medicine, Department of Social and Behavioral Sciences, Health Promotion and Behavior
Kyoto University Center for Medical Education and Internationalization / Population Health Policy Unit

**Statistician**
Name: Yan Luo
Affiliation: Kyoto University Center for Medical Education and Internationalization / Population Health Policy Unit, Assistant Professor

**19. Amendments and Revisions to the Research Protocol**

If amendments or revisions to this protocol are required, a modification request will be submitted to the ethics review committee for approval.

**20. Ethical Guidelines and Ethical Review**

This study will be conducted in accordance with the Declaration of Helsinki and the Ethical Guidelines for Life Science and Medical Research Involving Human Subjects. The study will be reviewed by the Ethics Committee of the Kyoto University Graduate School of Medicine / Faculty of Medicine / University Hospital and implemented with approval of the head of the research institution.

**21. Ownership of Research Outputs**

The research results belong to Kyoto University.

**22. References**

1 Shield KD, Rylett M, Gmel G, Gmel G, Kehoe-Chan TAK, Rehm J. Global alcohol exposure estimates by country, territory and region for 2005-a contribution to the Comparative Risk Assessment for the 2010 Global Burden of Disease Study. Addiction. 2013;108(5):912–22.

2 Boffetta P, Hashibe M, La Vecchia C, Zatonski W, Rehm J. The burden of cancer attributable to alcohol drinking. Int J Cancer. 2006;119(4):884–7.

3 Nelson DE, Jarman DW, Rehm J, Greenfield TK, Rey G, Kerr WC, et al. Alcohol-attributable cancer deaths and years of potential life lost in the United States. Am J Public Health. 2013;103(4):641–8.

4 Jarl J, Gerdtham UG. Time pattern of reduction in risk of oesophageal cancer following alcohol cessation-a meta-analysis. Addiction. 2012;107(7):1234–43.

5 Baan R, Straif K, Grosse Y, Secretan B, El Ghissassi F, Bouvard V, et al. Carcinogenicity of alcoholic beverages. Lancet Oncol. 2007;8(4):292–3.

6 Brooks PJ, Enoch M-A, Goldman D, Li T-K, Yokoyama A. The Alcohol Flushing Response: An Unrecognized Risk Factor for Esophageal Cancer from Alcohol Consumption. PLoS Med. 2009;6(3):e1000050.

7 Chen YC, Peng GS, Tsao TP, Wang MF, Lu RB, Yin SJ. Pharmacokinetic and pharmacodynamic basis for overcoming acetaldehyde-induced adverse reaction in asian alcoholics, heterozygous for the variant ALDH2*2 gene allele. Pharmacogenet Genomics. 2009;19(8):588–99.

8 Peng G, Wang M, Chen C, Luu S, Chou H, Li T, et al. Involvement of acetaldehyde for full protection against alcoholism by homozygosity of the variant allele of mitochondrial aldehyde dehydrogenase gene in Asians. Pharmacogenetics. 1999;9(4):463–76.

9 Luczak SE, Glatt SJ, Wall TL. Meta-analyses of ALDH2 and ADH1B and alcohol dependence in Asians: Examining models of influence. Psychol Bull. 2006;132(4):607–21.

10 Eng MY, Ph D, Luczak SE, Wall TL. ALDH2, ADH1B, and ADH1C in Asians: A literature review. Alcohol Res Heal. 2007;30(1):22–7.

11 Lewis SJ, Smith GD. Alcohol, ALDH2, and esophageal cancer: A meta-analysis which illustrates the potentials and limitations of a Mendelian randomization approach. Cancer Epidemiol Biomarkers Prev. 2005;14(8):1967–71.

12 Yang CX, Matsuo K, Ito H, Hirose K, Wakai K, Saito T, et al. Esophageal cancer risk by ALDH2 and ADH2 polymorphisms and alcohol consumption: Exploration of gene-environment and gene-gene interactions. Asian Pacific J Cancer Prev. 2005;6(3):256–62.

13 Yokoyama A, Kato H, Yokoyama T, Tsujinaka T, Muto M, Omori T, et al. Genetic polymorphisms of alcohol and aldehyde dehydrogenases, and drinking, smoking and diet in Japanese men with oral and pharyngeal squamous cell carcinoma. Carcinogenesis. 2002;28(4):865–74.

14 Roerecke M, Shield KD, Higuchi S, Yoshimura A, Larsen E, Rehm MX, et al. Estimates of alcohol-related oesophageal cancer burden in Japan: Systematic review and meta-analyses. Bull World Health Organ. 2015;93(5):329-338C.

15 Kaner E, Beyer F, Muirhead C, Campbell F, Pienaar E, Bertholet N, et al. Effectiveness of brief alcohol interventions in primary care populations. Cochrane Database Syst Rev. 2018;(2):1–248.

16 Substance Abuse and Mental Health Services Administration. Systems-Level Implementation of Screening, Brief Intervention, and Referral to Treatment. 2013.Available from: https://store.samhsa.gov/system/files/sma13-4741.pdf

17 Prochaska JO, Di Clemente CC. Transtheoretical therapy: Toward a more integrative model of change. Psychother Theory Res Pract. 1982;19(3):276–88.

18 Young MM, Stevens A, Galipeau J, Pirie T, Garritty C, Singh K, et al. Effectiveness of brief interventions as part of the Screening, Brief Intervention and Referral to Treatment (SBIRT) model for reducing the nonmedical use of psychoactive substances: A systematic review. Syst Rev. 2014;3(1):1–18.

19 Madras BK, Compton WM, Avula D, Stegbauer T, Stein JB, Clark HW. Screening, brief interventions, referral to treatment (SBIRT) for illicit drug and alcohol use at multiple healthcare sites: Comparison at intake and 6 months later. Drug Alcohol Depend. 2009 Jan;99(1–3):280–95.

20 Rastegar D, Fingerhood M. The American Society of Addiction Medicine Handbook of Addiction Medicine. New York, NY: Oxford University Press; 2015.

21 Hendershot CS, Otto JM, Collins SE, Liang T, Wall TL. Evaluation of a brief web-based genetic feedback intervention for reducing alcohol-related health risks associated with ALDH2. Ann Behav Med. 2010;40(1):77–88.

22 Gross ER, Zambelli VO, Small BA, Ferreira JCB, Chen CH, Mochly-Rosen D. A personalized medicine approach for Asian Americans with the aldehyde dehydrogenase 2∗2 variant. Annu Rev Pharmacol Toxicol. 2015;55:107–27.

23 Collins RL, Parks GA, Marlatt AG. Social determinants of alcohol consumption: The effects of social interaction and model status on the self-administration of alcohol. J Consult Clin Psychol. 1985;53(2):189–200.

24 Piumatti G, Aresi G, Marta E. A psychometric analysis of the Daily Drinking Questionnaire in a nationally representative sample of young adults from a Mediterranean drinking culture. J Ethn Subst Abuse. 2021;0(0):1–19.

25 Neighbors C, Dillard AJ, Lewis MA, Bergstrom RL, Neil TA. Normative misperceptions and temporal precedence of perceived norms and drinking. J Stud Alcohol. 2006;67(2):290–9.

26 Napper LE, Kenney SR, Lac A, Lewis LJ, LaBrie JW. A cross-lagged panel model examining protective behavioral strategies: Are types of strategies differentially related to alcohol use and consequences? Addict Behav. 2014;39(2):480–6.

27 Babor TF, Higgins-Biddle JC, Saunders JB, Monteiro MG. The Alcohol Use Disorders Identification Test: Guidelines for Use in Primary Care (WHO/MSD/MSB/01.6a). Gensva, Switzerland; 2001.Available from: http://whqlibdoc.who.int/hq/2001/WHO_MSD_MSB_01.6a.pdf

28 Reinert DF, Allen JP. The alcohol use disorders identification test: An update of research findings. Alcohol Clin Exp Res. 2007;31(2):185–99.

29 Sahker E, Lancianese D, Arndt S. Stability of the alcohol use disorders identification test in practical service settings. Subst Abuse Rehabil. 2017;8:1–8.

30 Kawada T, Inagaki H, Kuratomi Y. The alcohol use disorders identification test: Reliability study of the Japanese version. Alcohol. 2011;45(3):205–7.

31 Yokoyama T, Yokoyama A, Kato H, Tsujinaka T, Muto M, Omori T, et al. PROBLEM ORs - Alcohol Flushing, Alcohol and Aldehyde Dehydrogenase Genotypes, and Risk for Esophageal Squamous Cell Carcinoma in Japanese Men. Cancer Epidemiol Biomarkers Prev. 2003;12(11 II):1227–33.

32 Kevington S, Lotfy M, O’Connell K. The World Health Organization’s WHOQOL-BREF quality of life assessment: Psychometric properties and results of the international field trial A Report from the WHOQOL Group. Qual Life Res. 2004;13(2):299–310.

33 Clifton L, Clifton DA. The correlation between baseline score and post-intervention score, and its implications for statistical analysis. Trials. 2019;20(1):4–9.
