# Supplementary material for: Unguided web-based brief intervention with genetic risk education to reduce unhealthy alcohol consumption in Japan: Protocol for a randomized controlled trial
Source: PLoS One. 2026 Apr 17;21(4):e0347064. doi: 10.1371/journal.pone.0347064 (PMC13089686; doi:10.1371/journal.pone.0347064)
Supplement: S5 Table — (DOCX) [file pone.0347064.s005.docx]

**Table S5.** Informed consent document (translated from Japanese)

**Brief Intervention to Promote Healthy Behavior: Randomized Controlled Trial** **Informed Consent Document**

Date of Creation: May 30^th^, 2025

**1. About the Clinical Trial**

To maintain a healthy lifestyle, it is considered necessary for individuals to adopt seven health habits (breakfast, sleep, smoking, snacking, alcohol consumption, exercise, weight control). Currently, these are used as indicators of health habits, and efforts are being made to improve dietary habits, environmental factors, and physical constitution in individuals' lifestyles to enhance Quality of Life (QOL). A means that has recently gained attention for this purpose is Brief Intervention (BI). This study investigates whether viewing short videos as part of Brief Intervention can improve health.

**2. Study Title, Ethics Review, and Permission**

This study, "Brief Intervention to Promote Healthy Behavior: Randomized Controlled Trial," has been reviewed by the Ethics Committee of the Graduate School of Medicine, Faculty of Medicine, Kyoto University, and has been conducted with permission from the institutional head.

**3. Name of Research Institution and Principal Investigator**

**Principal Research Institution:** Kyoto University, National University Corporation

Principal Investigator: Ethan Sahker (Field of Health Promotion and Behavioral Science)

**Collaborating Research Institution:** Neo Marketing Co., Ltd.

Principal Investigator: Yoshiki Sakajiri

**4. Research Implementation Method, Period, and Participation Requirements** **Summary of Methods**

In this study, we will verify the effectiveness of Brief Intervention (BI) on a portal site among adults aged 20 and over and under 65 who understand Japanese. Your cooperation involves three specific tasks: first, accessing the web portal and participating in a brief survey; then, undergoing BI; and finally, responding to a follow-up survey three months later.

**Flow of Study**

1. Consent for Research Collaboration Participants who have listened to the explanation of this study and agreed to participate will access the web portal via QR code and complete a brief survey.
2. Randomization Participants who have given consent will be randomly assigned to either the intervention group or the control group.
3. Program Initiation You will watch videos on the portal site about improving health conditions. From survey to video viewing, the entire process will take about 30-45 minutes.
4. Follow-up at 1-, 2-, and 3-Months: A follow-up survey will be conducted 1, 2, and 3 months after completing the above steps.

**Electronic Consent**

On the portal site, you will first be asked, "Have you read the information document and agree to participate in this study?" Only those who select "Agree" will be able to participate in the study. Identity verification will be conducted by Neo Marketing Co., Ltd., and will require submission of an identity card with a photograph.

**Possibility of Halting the Research Program**

Even if the research program has already begun, if it is deemed inappropriate to continue the program for participants due to safety concerns or other reasons by the responsible individuals and principal investigator, the research program may be terminated. Please be aware of this possibility.

**Overall Research Period**

From July 9, 2025, to March 31, 2029

**5. Participants in the Study**

Estimated Number of Participants: 100

Eligibility Criteria for Participation in This Study:

1. Men and women aged 20 to under 65
2. No problems with reading, writing, and listening to Japanese
3. Access to an internet connection
4. Currently have an email account

Ineligible Participants for This Study:

1. Currently undergoing treatment for alcohol dependence or alcohol-related disorders
2. Have ever been diagnosed with or treated for cancer
3. Currently pregnant

**6. Burden on Participants and Expected Risks and Benefits**

Expected Benefits

1. Participants are expected to learn methods to improve their health. However, specific benefits from this study cannot be conclusively stated at this stage.

Expected Burden and Risks

1. Participating in the research program will require time for surveys and viewing videos as part of Brief Intervention.
2. Due to the randomization process, participants cannot choose which program they will be assigned to.
3. In this study, it is believed that the risk of health damage associated with the research will not increase compared to everyday activities such as general daily activities and internet use.

**7. Voluntariness of Study Participation**

Participation in this study is entirely voluntary. Even after receiving an explanation, you may refuse to participate without any disadvantage to yourself.

**8. Freedom to Withdraw from the Study**

Even after agreeing to participate in this study, you may withdraw from the study at any time. Your decision to withdraw will not result in any disadvantage to you.

**9. Disclosure of Research Information**

Details of this research plan will be published in academic journals written in English. In Japanese, they will be published on the website of the Graduate School of Medicine, Health Promotion and Behavioral Science Field, Kyoto University. Additionally, the overview and status of the clinical trial will be registered and disclosed on the University Hospital Medical Information Network Research Center's clinical trial registration system (UMIN-CTR).

**10. Handling of Research Results**

Results obtained from this trial will be presented at conferences and in medical journals. Furthermore, medical information obtained from this trial may be provided to or shared with third parties domestically and internationally, such as researchers, medical professionals, regulatory authorities, and related organizations.

**11. Potential Use of Information Obtained in Future Research**

Medical information obtained in this trial may be used (secondary use) for the advancement of medical and scientific research and for the improvement of public health related to maintaining and promoting people's health. However, such considerations are currently unplanned or scheduled for the future. Please note that specific details regarding which third parties in which countries the medical information obtained in this trial will be provided to or shared with, as well as information on measures taken to protect personal information by such third parties, cannot be specified at this time. Data with identifiable information removed will be registered and disclosed on the University Hospital Medical Information Network Case Data Repository (UMIN-ICDR) (https://www.umin.ac.jp/icdr/index-en.html). However, no information that can identify specific individuals, such as your name or address, will be reported.

**12. Handling of Personal Information**

All participants will be assigned an ID number, and all records will be managed under this ID number. Data transfer between the app and server is secured via SSL, and data is stored on secure servers. The management team regularly backs up data on secure servers. After the research period ends, data will be anonymized with the research identification code (a combination of letters, numbers, and symbols) and provided to or shared with third parties. Only the principal investigator will have access to the data. After the research period ends, data will be made unidentifiable, and all information that can identify individuals will be destroyed.

**13. Storage and Disposal Methods for Samples and Information**

Data will be collected and stored by Neo Marketing Co., Ltd. Individuals who have agreed to Neo Marketing's data storage policy and conditions before participating will become study participants. After data collection is complete, Neo Marketing Co., Ltd. will provide raw data to the principal investigator. These raw data will be stored on secure servers locked within Kyoto University's facilities, protected by passwords. Only the principal investigator will have access to the data.

**14. Funding Source and Conflict of Interest Regarding Research**

This clinical study is conducted with funding from the Japan Society for the Promotion of Science (JSPS) Grants-in-Aid for Scientific Research. This study does not receive funding from specific companies. Neo Marketing Co., Ltd., a collaborating research institution, will recruit study participants. Regarding conflicts of interest, appropriate scrutiny has been conducted by the Kyoto University Conflict of Interest Policy and Kyoto University Conflict of Interest Management Regulations through the Kyoto University Clinical Research Conflict of Interest Review Committee. Intellectual property rights arising from the research belong to Kyoto University.

**15. Response to Consultations Regarding Research and Contact Information**

Inquiries regarding this study will be accepted by the study secretariat. If you have any concerns or uncertainties, please contact us at the following: <Study Secretariat> Postal code 606-8501 Kyoto City, Sakyo-ku, Yoshida Konoe-cho Graduate School of Medicine, Health Promotion and Behavioral Science Field, Kyoto University Phone number: 075-753-9491 Hours of operation: Monday to Friday (9:00-17:00) Name: Ethan Sahker (Assistant Professor, Health Promotion and Behavioral Science Field)

<Contact for Complaints and Consultations> Postal code 606-8501 Kyoto City, Sakyo-ku, Yoshida Konoe-cho Graduate School of Medicine, General Affairs Planning Division, Research Promotion Section Phone number: 075-753-9301 E-mail: 060kensui@mail2.adm.kyoto-u.ac.jp

**16. Economic Burden and Existence of Compensation**

For the time and effort involved in participating in this study, participants will receive a gift card or equivalent payment of ¥700 upon completion of the initial survey and video viewing. Additionally, ¥200 will be paid upon completion of the surveys at 1, 2, and 3 months later. An additional ¥1,000 will be paid to those who complete all processes. Therefore, total compensation can reach up to ¥2,300. However, communication costs incurred in participation will be borne by the participants.

**Electronic Explanatory Document**

**Participant Consent to Participate in the Study**

By selecting “I agree” in response to the first question on the survey form—“Having read the explanation document, do you agree to participate in this study?”—you indicate your consent to participate in this study.

Please be sure to read and agree to the participant terms and privacy policies of Neo Marketing, as they are related to the management of your personal ID.
