# Supplementary material for: Unguided web-based brief intervention with genetic risk education to reduce unhealthy alcohol consumption in Japan: Protocol for a randomized controlled trial
Source: PLoS One. 2026 Apr 17;21(4):e0347064. doi: 10.1371/journal.pone.0347064 (PMC13089686; doi:10.1371/journal.pone.0347064)
Supplement: S6 Table — (DOCX) [file pone.0347064.s006.docx]

**Table S6.** Statistical Analysis Plan

### **STATISTICAL ANALYSIS PLAN**

**Unguided Web-Based Brief Intervention with Genetic Risk Education to Reduce Unhealthy Alcohol Consumption: Protocol for a Randomized Controlled Trial**

**Version 1.0**

**2025/07/9**

### **1. Study Overview**

#### 1.1. Objectives

#### The present study will test the efficacy of a web-based brief intervention (BI), using personalized genetic cancer risk education to reduce alcohol consumption for participants who screen positive for probable possession of the ALDH2*2 allele.

#### 1.2. Participants

Subjects will include Japanese adults in the general population who are of drinking age (20 years old), regularly drink alcohol, screen positive for the probable *ALDH2*2* genotype, not currently pregnant, not currently diagnosed with cancer, and not currently receiving alcohol treatment.

*1.2.2. Inclusion Criteria*

1. 20 to 64 years of age
2. At least 4 drinks per week
3. Positive screening for probable *ALDH2**2 allele

*1.2.3. Exclusion*

1. Current alcohol use treatment
2. Lifetime cancer diagnosis
3. Pregnant

#### 1.3 Intervention

- **Intervention group**: The BI consists of an animated educational video with a host medical doctor avatar voiced by a professional voice-over artist. It provides spoken information, animated content, and graphical depictions of graphs, charts, and genetic processes. Education is provided on the ALDH2 enzyme, the influence of ALDH2 genetic variations on alcohol metabolism, the relationship between ALDH2*2 genotype and esophageal cancer risk, and the risks of continued use. The video will last approximately 6 minutes, and delivery of the intervention content is based on the FRAMES approach. FRAMES includes Feedback, Responsibility, Advice, a Menu of options, Empathy, Supports Self-Efficacy.
- **Control group**: The control group will receive a sham video that describes the importance of quality of life (QoL) and health, description of QoL concepts, ways to change health behaviors to improve QoL. Alcohol consumption is not mentioned. The control sham-intervention consists of an animated educational video with a host medical doctor avatar voiced by a professional voice-over artist. It provides spoken information, and animated content will last approximately 6 minutes.

#### 1.4. Study Design

Double-blind (blind to study aims), parallel group, randomized controlled trial.

#### 1.5. Target Sample Size

A total of 64 participants, with 32 in the intervention group and 32 in the control group.

### **2. Analysis Samples**

#### 2.1. Efficacy Analysis Sample (FAS)

The full analysis set (FAS) will include the largest sample for analysis based on the intention-to-treat principle, excluding participants who:

- Withdraw consent and disallow the use of their data after enrollment.
- Have no efficacy-related data available after starting the intervention.

#### 2.2. Safety Analysis Sample (SAS)

The safety analysis sample will include all randomized participants, including those who discontinue or drop out of the study.

**3. Outcome Measures**

#### 3.1. Primary Outcome

#### Endpoint past 4-week alcohol use quantity at 3-months post-randomization. Alcohol use quantity will be measured in number of standard drinks with the Daily Drinking Questionnaire (DDQ).

#### 3.2. Secondary Outcomes

1. Endpoint past 4-week alcohol use quantity measured in number of standard drinks with DDQ at 1- and 2-months post-randomization
2. Alcohol use quantity measured in grams converted from DDQ at 1-, 2-, and 3-months post-randomization
3. Within group change in past 4-week alcohol use quantity measured in number of standard drinks with DDQ at 1-, 2-, and 3-months post-randomization
4. Severity at 1-, 2-, and 3-months post-randomization measured with the Alcohol Use Disorders Identification Test (AUDIT)
5. Readiness to change: Modeled on the Transtheoretical Model of Change with questions taken from the Japanese National Health and Nutrition Survey measured at 1-, 2-, and 3-months post-randomization.
6. Health knowledge retention: a single question asking if participants recall their ALDH2 allele measured at 1-, 2-, and 3-months post-randomization
7. Quality of Life measured with the Japanese version of the WHOQOL-BREF at 1-, 2-, and 3-months post-randomization
8. Participant Satisfaction: 5 questions about program content measured at treatment completion

### **4. Statistical Analysis**

#### Primary Outcome

The primary analysis will focus on the FAS sample using the primary outcome—alcohol use quantity in numbers of standard drinks as measured by the DDQ at the 3-month follow-up. A Generalized Linear Mixed Model (GLMM) will be used, assuming a negative binomial distribution to address possible overdispersion in the count data. GLMM will incorporate all available repeated measures to account for missing outcome data by modeling within-subject correlations over time under the missing at random (MAR) assumption. The model will include fixed effects for treatment group (intervention vs. control), time point (months 1, 2, and 3), and their interaction, as well as random effects for both the intercept and the slope for time at participant level to account for individual variability in baseline outcome levels and temporal trends. Time will be used as a categorical variable. If model convergence is not achievable—particularly due to the limited sample size—simplified models will be employed. First, a model will be fitted with time treated as a categorical variable but including only a random intercept to reduce complexity. Alternatively, time may be modeled as a continuous linear variable, allowing for a random slope to be retained while simplifying the fixed effects structure. In addition, model fit and distributional assumptions will be evaluated by inspecting residuals and testing for overdispersion. If substantial violations are identified, alternative model specifications (e.g., Poisson, zero-inflated models) may be considered. The model will estimate marginal means (EMMs) for each group at the 3-month follow-up and report the difference between groups with corresponding 95% confidence intervals (CIs). Statistical significance will be determined using a two-sided alpha level of 0.05.

A sensitivity analysis using the SAS sample will be conducted for the primary outcome with only complete follow-up cases included. We will conduct subgroup analysis to explore potential interactions between the group and the following pre-specified factors: age, sex, severity, health knowledge retention. These factors will be included as covariates in the model, along with their interaction terms with the intervention group. The p-value for each interaction term will be estimated.

#### Secondary Outcomes

Secondary outcomes measured with discrete count variables will be analyzed in the same method as the primary outcome. Continuous secondary outcomes including alcohol use in grams, readiness to change, and quality of like will be analyzed using a mixed model for repeated measures (MMRM) to account for within-subject correlations over time. The MMMR model will include fixed effects for treatment group (intervention vs. control), time (months 1, 2, and 3), and the treatment-by-time interaction as a covariate. The covariance structure for the repeated measures will initially be specified as unstructured. However, if convergence cannot be achieved, simpler covariance structures such as compound symmetry or autoregressive (AR(1)) may be evaluated using model fit criteria (e.g., Akaike Information Criterion, Bayesian Information Criterion). Binary secondary outcomes, such health knowledge retention, will be analyzed using a GLMM with a binomial distribution. The model specification will follow the same structure as that used for the primary outcome analysis. Participant satisfaction, will be assessed only in the intervention group, will be summarized descriptively.

#### Statistical Software

All analyses will be conducted using R version 4.2.3. GLMM models will use the *glmmTMB* and lme4 packages, and MMRM models using the mmrm and emmeans packages.
